# *Lilium liangiae*, a new species in the genus *Lilium* (Liliaceae) that reveals parallel evolution within morphology

**DOI:** 10.3389/fpls.2024.1371237

**Published:** 2024-03-27

**Authors:** Yumei Yuan, Yundong Gao

**Affiliations:** ^1^ CAS Key Laboratory of Mountain Ecological Restoration and Bioresource Utilization and Ecological Restoration and Biodiversity Conservation Key Laboratory of Sichuan Province, Chengdu Institute of Biology, Chinese Academy of Sciences, Chengdu, China; ^2^ College of Life Sciences, University of Chinese Academy of Sciences, Beijing, China

**Keywords:** *Lilium liangiae*, *Nomocharis*, *Lilium*, phylogeny, morphology, ITS, complete chloroplast, comparative genomics

## Abstract

The former genus *Nomocharis*, which has been merged as a clade within the genus *Lilium* (Liliaceae), represents one of the most complicated and unclear groups included in the latter. Research on members of the Nomocharis clade has been quite limited due to the sampling difficulties caused by its selective environmental preferences. In this study, we propose a new species within this clade, *Lilium liangiae*, as a further bridge connecting the former genus *Nomocharis* with other members of the genus *Lilium.* We conducted morphological clustering, phylogenetic, and comparative genomics analyses of nuclear internal spacers and the newly generated complete chloroplast genome, in conjunction with previously published sequences, and performed ancestral state reconstruction to clarify the evolutionary pattern of important traits in *Lilium*. The clustering results of 38 morphological traits indicated that the new species is allied to Nomocharis, further increasing the morphological polymorphism in the latter. The phylogenetic results and morphological clustering both supported *L. liangiae* belonging to the subclade Ecristata in Nomocharis, its closest affinity being *Lilium gongshanense*. Inconsistencies in phylogenetic relationships were detected between nuclear and plastid datasets, possibly due to ancient hybridization and ongoing introgression. Comparative genomics revealed the conservation and similarity of their chloroplast genomes, with variations observed in the expansion and contraction of the IR regions. A/T and palindromic repeat sequences were the most abundant. Seven highly variable regions (Pi≥0.015) were identified as potential molecular markers based on the chloroplast genomes of 47 species within *Lilium*. Both nuclear and plastid genes exhibited very low variability within the Nomocharis clade, contrasting with their highly variable morphological appearance. The ancestral state reconstruction analysis suggests that the campanulate flower form, as in *L. liangiae*, arose at least three times within the genus *Lilium*, revealing parallel evolution in the latter. Overall, this study adds important genetic and morphological evidence for understanding the phylogenetic relationships and parallel evolution patterns of species within the genus *Lilium*.

## 1 Introduction

*Lilium* Linnaeus (Linnaeus, 1753) is a genus consisting of approximately 115~130 species found in temperate and alpine regions of the Northern Hemisphere (Liang and Tamura, 2000) and 118 taxa recorded by Plants of the World Online (POWO, 2024). Its distribution is particularly notable in eastern Asia, especially southwest China (Liang, 1995; Liang and Tamura, 2000), where a diverse array of lilies with striking and variable perianth shapes and colors can be observed, many of which possess considerable horticultural value (Liang and Tamura, 2000). *Nomocharis* Franchet (Franchet, 1889) was initially established as an independent genus close to *Lilium* and *Fritillaria* in the late 19th century. After much debate about the status of *Lilium* and *Nomocharis* (Balfour, 1918; Evans, 1925; Sealy, 1950, 1983; Liang, 1984), the latter was eventually accommodated and nested in *Lilium* as a subgenus clade based upon molecular phylogenetics (Nishikawa et al., 1999; Peruzzi et al., 2009; Gao et al., 2013; Du et al., 2014; Gao et al., 2015; Gao and Gao, 2016; Huang et al., 2018; Li et al., 2022a; Zhou et al., 2023). As one of the most complicated and unclear subgroups in *Lilium*, previous studies have provided us with a superficial understanding of its position within the genus and the possible processes of speciation that were involved (Gao et al., 2012, 2013, 2015; Zhou et al., 2023), but the detailed phylogenetic framework and evolutionary mechanisms are not clear due to limited sampling.

**Figure 1:**
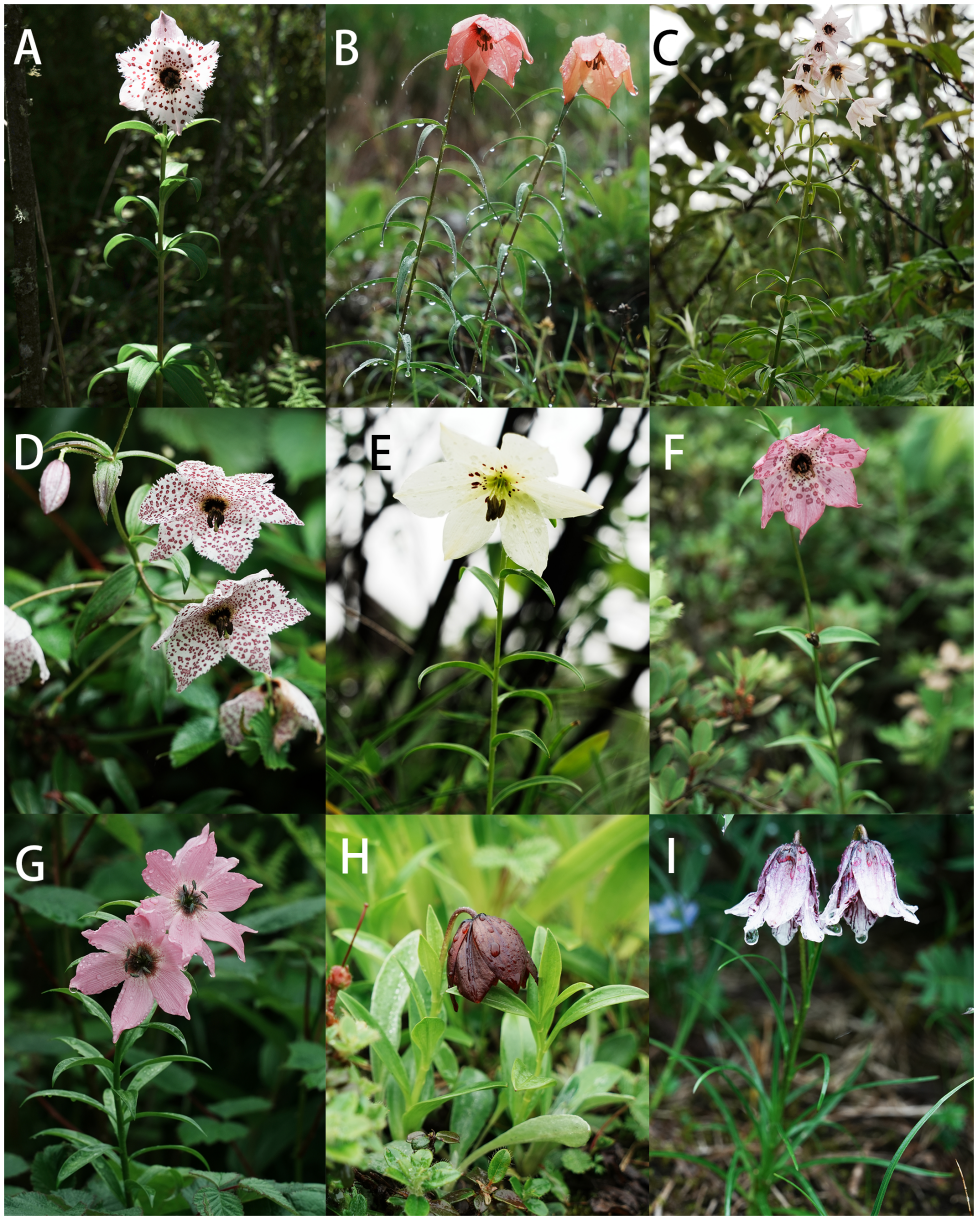
Field pictures of some Nomocharis clade species. **(A)** *Lilium pardanthinum*. **(B)** *Lilium basilissum*. **(C)** *Lilium sealyi*. **(D)** *Lilium meleagrinum*. **(E)** *Lilium gongshanense*. **(F)** *Lilium apertum*. **(G)** *Lilium saluenense*. **(H)** *Lilium souliei*. **(I)** *Lilium yapingense*.

The name “Nomocharis” is composed of the Greek “nomos”, which means meadow or pasture, and “charis”, which means outward grace or loveliness (**Figure 1**; Woodcock and Stearn, 1950). Franchet named it in this way to recognize the group’s outstanding beauty as well as its typical habitat. With its raceme of white or pink, flat, plate-like flowers variously sprinkled with darker spots and speckles, held above handsomely whorled leaves in several layers, *Lilium pardanthinum* (Franch.) Y.D.Gao (formerly *Nomocharis pardanthina* Franch.) immediately became a popular and coveted horticultural subject after its initial publication. Swaying in the wind on the plateau, typical members of *Nomocharis* exhibit a distinctive saucer-shaped or campanulate corolla and thrive on damp slopes (Gao and Gao, 2016). The group’s distribution is narrow and limited to the Hengduan (H-D) Mountains of southwestern China, the eastern Qinghai–Tibetan Plateau (QTP), and adjacent Myanmar and India (Sealy, 1950, 1983; Liang, 1984; 1995; Liang and Tamura, 2000). These regions were formed relatively recently and underwent quite severe changes in geographic features, and this led previous authors to speculate upon the likelihood of a comparatively short process of speciation and evolution in Nomocharis clade taxa contemporaneously with the uplift of eastern Tibet and the H-D Mountains (Liang, 1984; 1995; Gao et al., 2015) and, therefore, that the group had a recent origin.

During the process of rapid diversification and adaptation to the environment, the Nomocharis clade exhibits the contradiction of high divergence in morphology but with low genetic distancing, which has resulted in significant morphological differences compared to typical lily species (Gao et al., 2015; Zhou et al., 2023). Such rapid diversification had already been detected in other groups such as *Ligularia*–*Cremanthodium*–*Parasenecio* complex (Asteraceae), *Rheum* (Polygonaceae), and *Rhodiola* (Crassulaceae) (Liu et al., 2006; Sun et al., 2012; Zhang et al., 2014) (for further groups displaying such a pattern, see Gao et al., 2015, **Table 1**). Gao et al. (2015) pointed out that the morphological differences between typical *Nomocharis* (flat flowers) and its subclade, the Non-Nomocharis Lilies (campaniform flowers), resulted from the mountain building causing habitat specialization. Comparatively, the nodding bell-shaped corolla was more effective in protecting internal reproductive structures, adapting to the torrential rain and strong winds of alpine meadows, despite a certain reduction in pollination efficiency (Percival, 1955; Mao and Huang, 2009; Lawson and Rands, 2019). In contrast, flat-shaped flowers growing in the understory or among shrubs experienced relatively minor impacts from rain and wind, and at the same time, their larger open corollas enabled higher pollination efficiency (Gao et al., 2015). This situation led to significant morphological differences among *Lilium* species (especially in *Nomocharis*) with close genetic relationships, while species with similar morphologies, as a result of comparable environmental pressures, may actually have distant relationships.

**Table 1:** Characteristics of the newly generated complete chloroplast genomes for *Lilium*.

Sample code	Species name	Size (bp)				Number of genes/all (unique)					Accession number in GenBank	Sample location
		Total	LSC	IR	SSC	Total	Protein coding genes	tRNA	rRNA	Total GC%		
NG19	*Lilium gongshanense*	151,969	81,620	26,440	17,469	136 (115)	84 (78)	38 (30)	8 (4)	37.00%	OR353697	China. Yunnan: Gongshan
NG9	*L. gongshanense*	151,969	81,620	26,440	17,469	136 (115)	84 (78)	38 (30)	8 (4)	37.00%	OR353696	China. Yunnan: Gongshan
NS13	*Lilium saluenense*	152,046	81,698	26,440	17,468	136 (115)	84 (78)	38 (30)	8 (4)	37.00%	OR353700	China. Yunnan: Gongshan
NS14	*L. saluenense*	152,046	81,698	26,440	17,468	136 (115)	84 (78)	38 (30)	8 (4)	37.00%	OR353701	China. Yunnan: Gongshan
gyd1411_1	*Lilium liangiae*	152,548	81,940	26,536	17,536	136 (115)	84 (78)	38 (30)	8 (4)	37.00%	OR353690	China. Xizang: Bomi
gyd1411_2	*L. liangiae*	152,547	81,939	26,536	17,536	136 (115)	84 (78)	38 (30)	8 (4)	37.00%	OR353691	China. Xizang: Bomi
gyd1411_3	*L. liangiae*	152,547	81,939	26,536	17,536	136 (115)	84 (78)	38 (30)	8 (4)	37.00%	OR353692	China. Xizang: Bomi
gyd1408	*Lilium medogense*	152,357	82,002	26,415	17,525	136 (115)	84 (78)	38 (30)	8 (4)	37.00%	OR353688	China. Xizang: Motuo
gyd1420	*Lilium souliei*	152,324	81,806	26,451	17,616	136 (115)	84 (78)	38 (30)	8 (4)	37.00%	OR353693	China. Xizang: Chayu
gyd1406	*Lilium paradoxum*	151,839	81,469	26,421	17,528	136 (115)	84 (78)	38 (30)	8 (4)	37.00%	OR353687	China. Xizang: Milin
NM18	*Lilium meleagrinum*	152,437	81,864	26,520	17,533	136 (115)	84 (78)	38 (30)	8 (4)	37.00%	OR353699	China. Xizang: Bomi
NM13	*L. meleagrinum*	152,452	81,878	26,520	17,534	136 (115)	84 (78)	38 (30)	8 (4)	37.00%	OR353698	China. Yunnan: Gongshan
GYD344_2	*Lilium apertum*	152,469	81,890	26,515	17,549	136 (115)	84 (78)	38 (30)	8 (4)	37.00%	OR350450	China. Yunnan: Shangri-la
GYD344_3	*L. apertum*	152,470	81,891	26,515	17,549	136 (115)	84 (78)	38 (30)	8 (4)	37.00%	OR353684	China. Yunnan: Shangri-la
GYD0445	*Lilium sealyi*	152,023	81,877	26,428	17,290	136 (115)	84 (78)	38 (30)	8 (4)	37.00%	OR350449	China. Yunnan: Lushui
GYD2021002	*Lilium pardanthinum*	151,735	81,608	26,432	17,263	136 (115)	84 (78)	38 (30)	8 (4)	37.00%	OR353694	China. Yunnan: Lijiang
GYD2021003	*Lilium yapingense*	152,035	82,034	26,357	17,287	136 (115)	84 (78)	38 (30)	8 (4)	37.00%	OR353695	China. Yunnan: Fugong
gyd1392	*Lilium nepalense*	152,915	82,472	26,507	17,429	136 (115)	84 (78)	38 (30)	8 (4)	37.00%	OR353686	China. Xizang: Jilong
gyd1410	*Lilium wardii*	152,270	82,247	26,314	17,395	134 (114)	84 (78)	38 (30)	8 (4)	37.00%	OR353689	China. Xizang: Bomi
gyd1384	*Lilium nanum*	152,859	82,052	26,888	17,031	136 (115)	84 (78)	38 (30)	8 (4)	37.00%	OR353685	China. Xizang: Dingjie
GXF18899	*Lilium lophophorum*	152,397	82,032	26,436	17,493	134 (114)	84 (78)	38 (30)	8 (4)	37.00%	OR350448	China. Sichuan: Xiaojin

Closely related species that exhibit similar or identical appearances are summarized as having undergone parallel evolution or independent evolution of a special characteristic in a special group (Simpson, 1961; Haldane, 1990; Arendt and Reznick, 2008). This phenomenon has been detected in the evolution of floral shape in the genus *Lilium*, where trumpet-flowered members are found spread across several monophyletic clades, rather than clustering into a single clade as in the traditional subgeneric treatment (e.g., clades Liriotypus, Leucolirion, and Pseudolirium). This parallel evolution of floral appearance in *Lilium* was found to be a result of adaptation to comparable pollinators in similar environments (Liu et al., 2019). Within the Nomocharis clade, there are multiple features that exhibit this pattern, including floral appearance (flat, open, saucer-shaped, and campanulate), posture (horizontal and nodding), and leaf arrangement (alternate or whorl-leaved). These variable features, combined with differences in plant size, have caused significant challenges in the taxonomy of the Nomocharis clade. In the past, members of this clade had been assigned to different subgeneric sections, leading to much controversy.

The complicated relationships within the Nomocharis clade are also a consequence of ongoing gene flow. A previous study (Gao et al., 2020) revealed that interspecific introgression via hybridization is quite common in these sympatric lilies. In contrast, while such introgression prevails, the identities of each species involved remain quite distinct. The phenomena observed indicate that hybridization is not the result of their shared morphology but rather contributes to nuclear–cytoplasmic conflicts within phylogenetic relationships. The genetic linkage of large chromosomes, coupled with the effects of environmental filtering, results in extremely rare hybridization speciation events among *Lilium* species with generally low genetic distances. Amid ongoing gene flow, certain parapatrically distributed lilies also maintained their boundaries through some biological factors such as differences in phenology [e.g., *Lilium gongshanense* Y.D.Gao & X.J.He, *Lilium meleagrinum* (Franch.) Y.D.Gao, and *Lilium saluenense* (Balf. f.) S.Y.Liang; Gao et al., 2020]. The evolution of *Lilium* species, in terms of both genetics and morphology, unfolded asynchronously, primarily driven by the environment, which further contributed to the obscuring of species boundaries. Summing up the situation above, we should integrate diverse information, attempting to identify a suitable pattern to comprehensively review and adeptly manage relationships among *Lilium* species.

In this study, after decades of study of the lilies in southwestern China, we propose the new species, *Lilium liangiae*, a typical *Lilium*-like species with campanulate-shaped nodding flowers, as a further bridge to connect the former genus *Nomocharis* with the remainder of the genus *Lilium*, seeking to reveal the underlying consistency beneath the surface of morphological diversity. Through comparative genomic analysis of the entire Nomocharis clade and an improved phylogenetic framework, we aim to further elucidate the complex nature of the genus *Lilium* in a specific habitat. By integrating the characteristics of this new taxon, we further review the entire *Lilium* genus and propose new perspectives on the challenges in its classification. Most importantly, we seek to provide guidance on prioritizing the conservation of those species that face the impact of human activities and the risk of genetic extinction due to introgression.

## 2 Materials and methods

### 2.1 Plant material

In this study, leaf materials of 21 species, mainly in the Nomocharis clade and supplemented with representatives of the remainder of *Lilium*, were collected from the field and temporarily preserved in silica gel (**Table 1**). Voucher specimens were deposited in the Herbarium of the Chengdu Institute of Biology (CDBI).

The subordinate classification employed in this study is derived from Comber (1949), Liang (1980), and our previous studies (Gao et al., 2013, 2015). The selection of other sequences in phylogenetic analysis is grounded in prior studies (Gao et al., 2013, 2015), including representative species selected from each clade (**Supplementary Tables S1**, **S2**). Furthermore, based on the structure of the phylogenetic tree in this study, 23 representative species (**Supplementary Table S3**) were selected for comparative analysis of chloroplast genomes.

### 2.2 Morphological analysis

In order to determine the position of the new species *L. liangiae*, in terms of morphology, within the entire genus *Lilium*, we summarize a total of 38 representative and important quality traits by consulting Watanabe et al. (2021), *Flora of China* (Liang, 1980; Liang and Tamura, 2000), *Flora of North America* (http://floranorthamerica.org/Lilium), and online databases (high-resolution digit images from Chinese Virtual Herbarium, Plant Photo Bank of China and Wild Lilies Photo Gallery; bdlilies.com). We employed these traits, including features of flowers, leaves, and bulbs, and integrating important botanical classification features within the genus, with the aim of providing as comprehensive a description as possible for each clade. We used 1, 0 to record the presence or absence of qualitative trait data for the eight traditional subgeneric classifications (Comber, 1949; Liang, 1984); for one new clade, Nomocharis (Gao and Gao, 2016), and the newfound taxon *L. liangiae*, we performed hierarchical clustering of morphological data using OriginPro v2023 (https://www.originlab.com/) to explain the similarity of morphology among these groups. It is worth noting that when conducting the subdivision within *Lilium*, we also took into account differences in the classification proposed by Watanabe et al. (2021) and created a corresponding table (**Supplementary Table S4**). Due to the difficult delineation of certain morphological features of *Lilium nepalense* within *Nomocharis* (see Discussion section), we did not include its characteristics in the morphological clustering analysis.

### 2.3 DNA extraction, amplification, and sequencing

Genomic DNA was extracted from silica gel-dried leaves using a modified cetyltrimethylammonium bromide (CTAB) protocol based on modified Doyle’s method (Doyle and Doyle, 1987). Paired-end sequencing libraries were constructed using insert sizes of approximately 350 bp. The sequencing was performed using the DNBseq-4000 platform at the Beijing Genomics Institute (BGI; Shenzhen, China).

### 2.4 Chloroplast genome, ITS assembly, and annotation

Approximately 13 Gb of raw data were filtered and evaluated using fastp v0.23.2 (Chen et al., 2018) and FastQC (https://www.bioinformatics.babraham.ac.uk/projects/fastqc/) with default parameters. The sequence of chloroplast and internal transcribed spacer (ITS) were then assembled from clean data using GetOrganelle v1.7.6.1 (Jin et al., 2020). Chloroplast sequences of correct orientation were selected through multiple sequence alignment using Mafft v7 (https://mafft.cbrc.jp/alignment/software/; Katoh et al., 2019). Chloroplast genomes were annotated, and manual corrections were made using Geneious Prime v2023.1.2 (Biomatters Ltd., Auckland, New Zealand) based on the plastome of *L. gongshanense* (NC_052787) and *L. pardanthinum* (MG704135). The determination of ITS boundaries is based on alignment with previously published *Lilium* genus sequences (**Supplementary Table S1**), and all ITS sequences were analyzed using MEGA v11.0 (Tamura et al., 2021). The chloroplast genome map was generated online using the OrganellarGenomeDRAW tool (https://chlorobox.mpimp-golm.mpg.de/OGDraw.html; Greiner et al., 2019).

### 2.5 Evolutionary and phylogenetic analysis

To clarify the genetic position of the putative new taxon and indeed the whole Nomocharis clade in *Lilium*, the phylogenetic relationships were reconstructed based on both whole chloroplast genomes and ITS datasets. As for the two datasets (**Table 1**; **Supplementary Tables S1**, **S2**, **S5**), the sequence alignments were generated using MAFFT v7. Filtering of ambiguously aligned sites in alignment results of chloroplast genomes was performed using Gblocks v0.9b (Talavera and Castresana, 2007) with default parameters. The nucleotide substitution models were estimated using ModelTest-NG v0.1.7 (Darriba et al., 2020). Phylogenetic trees were inferred using maximum likelihood (ML) and Bayesian inference (BI). The ML trees were inferred using RAxML-NG v1.2.0 (Kozlov et al., 2019) with GTR+I+G4 and 1,000 bootstrap. The BI analyses were performed using MrBayes v3.2 (Ronquist et al., 2012) with the nucleotide substitution model GTR+G+I (lset nst=6 rates=invgamma). For each analysis, the posterior probability was estimated with two independent Markov chain Monte Carlo (MCMC) chains (10 million generations) with the preliminary 25% of sampled data discarded as burn-in.

### 2.6 Ancestral state reconstruction

To reveal the pattern of parallel evolution and its implications in the genus *Lilium*, four important traits were further selected to reconstruct the ancestral states, including floral appearance and posture, leaf arrangement, and habitat altitude. Floral and leaf traits might be influenced by complex environmental factors (e.g., moisture and illumination, which are correlated with altitude in most cases), thus resulting in parallel evolution while adapting to similar habitats (Gao et al., 2015). In particular, floral appearance and orientation have previously served as key traits for distinguishing the species in the Nomocharis clade from other lilies; therefore, they are quite important characteristics in the subgeneric delimitations. To reconstruct ancestral character states, different types for each trait were encoded using alphabet letters (A, B, C, etc.; see explanations in **Supplementary Tables S6**, **S7**). The more informative chloroplast genome BI tree was selected for ancestor state reconstruction using the Bayesian Binary method (BBM) in RASP (Yu et al., 2015). The tree was simplified using TreeGraph 2.0 (Stöver and Müller, 2010), and ancestral state reconstructions were carried out in RASP with 1,000,000 MCMC generations and the F81 + G model.

### 2.7 Chloroplast genome repeat sequence analysis and molecular marker identification

The MISA online website (https://webblast.ipk-gatersleben.de/misa; Beier et al., 2017) was used to perform simple sequence repeat (SSR) detection. The parameters were adjusted as follows: mononucleotides (10), dinucleotides (five), trinucleotides (four), tetranucleotides (three), and pentanucleotides and hexanucleotides (both three). Long repeats of forward, reverse, palindromic, and complementary sequences in the chloroplast genome were detected using REPuter (https://bibiserv.cebitec.uni-bielefeld.de/reputer; Kurtz et al., 2001), with a minimal repeat size of 30 bp and a Hamming distance of 3. Identification of molecular markers was first conducted by aligning chloroplast genome sequences using the MAFFT v7, and identification was carried out using DnaSP v6.12.03 (Rozas et al., 2017) under the conditions of window length of 600 bp and step size of 200 bp. Sequences used for analysis corresponded to the phylogenetic tree, with one sequence selected per species. Gene order arrangements were analyzed using the Mauve software (Darling et al., 2004), employing the progressive Mauve algorithm, and mVISTA (Frazer et al., 2004) was utilized to visually align the chloroplast genome with *L. gongshanense* as the reference. The plastome junction was analyzed using IRscope (Amiryousefi et al., 2018) to visualize the expansion and contraction of the boundaries of inverted repeats (IRs).

## 3 Result

### 3.1 Taxonomic treatment

**Figure 2:**
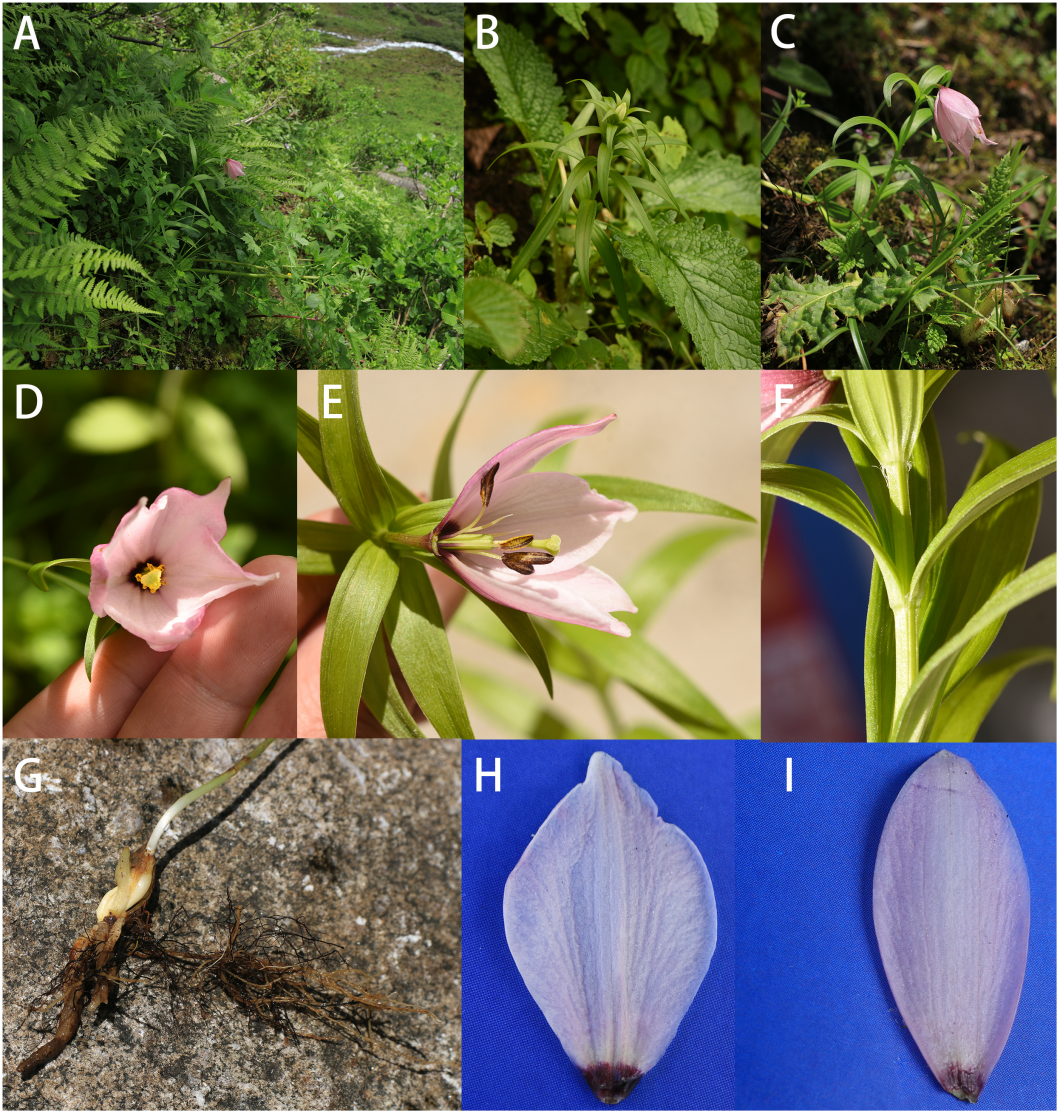
Morphological characteristics of *Lilium liangiae.* **(A)** Habitat. **(B, C)** Plant. **(D)** Flower. **(E)** Anatomical view. **(F)** Tufted hairs. **(G)** Bulb. **(H)** Inner tepal. **(I)** Outer tepal.

*L. liangiae* Y. M. Yuan et Y. D. Gao **sp. nov.** (**Figure 2**)

Type:—CHINA. Xizang: Linzhi City, Bomi County, elevation ca. 3,500 m, 3 July 2022, *GYD1411* (holotype: CDBI0286870; isotype: CDBI0286889).

At first glance, the new species bears a slight resemblance to *Lilium mackliniae* in terms of perianth morphology. Both have bell-shaped nodding flowers with petals curving upward at the end, and the petal edges transition from white to a deeper pink toward the center. Nonetheless, they significantly differ in overall plant morphology and leaf shape, with *L. liangiae* being shorter and smaller, typically bearing only one flower.

Perennial herbs, ca. 45-cm tall. Bulbs oblong, 2.6 × 1.2 cm, scales white without joint. Stem glabrous, striate, 34.7–41.6 cm. Stem rooting. Leaves cauline, alternate, linear to lanceolate, 5.3–7.1 × 0.5–0.9 cm. A pair of tufted hairs at the base of the leaf. Flowers nodding. Perianth is campanulate-shaped and pinkish, the edges of petals are white, with no spots or blotches, and occasionally one to two purple spots appear at the base. Tepals 6, outer oblong-oval, 2.5–3.3 × 1.0 cm, margin entire; inner, ovate-suborbicular, 2.4–3.2 × 1.5 cm, margin slightly erose; nectary possesses 2, purplish black, ridges of tissue arranged in a fan shape. Stamens 6, filaments, slender cylinder subulate at apex, ca. 9 mm long, bearing a yellow anther with black blotches ca. 5 × 1 mm. Ovary ca. 8 mm, style clavate, ca. 7 mm with trilobed stigma, 2 mm long.

Known only from Bomi County, Linzhi City (the precise location is withheld for protection and conservation reasons). The number of individuals is extremely low, and only four to five mature individuals can be found. It is critically endangered (CR; IUCN, 2022), a “plant species with extremely small populations”, demanding urgent protection. The epithet commemorates the Chinese botanist Liang Song-Yun (梁松筠) for her momentous contribution to our knowledge and understanding of the genera *Nomocharis* and *Lilium* in China.

### 3.2 Morphological analysis

**Figure 3:**
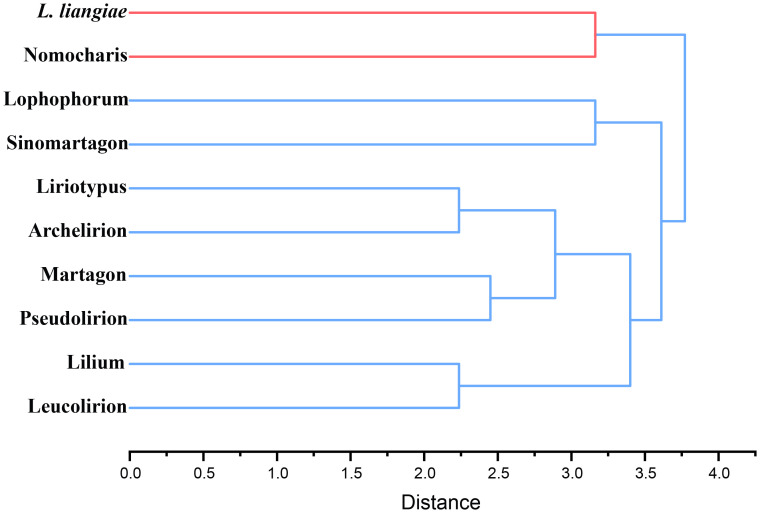
Hierarchical clustering of 10 groups of *Lilium* species based on morphological characteristics.

In order to quantify the morphological characteristics of this subgenus clade within the genus *Lilium*, we selected 38 traits, including features of the bulbs, roots, flowers, and leaves, and objectively defined them using a binary system (1, 0), indicating the presence or absence (**Supplementary Table S8**). We included 10 groups in hierarchical clustering, which we mainly divided into two clusters with *Nomocharis* and *L. liangiae* as one cluster and the other made up of the remaining groups that could be further subdivided into two clades: one of which included Lophophorum and Sinomartagon, and the other Liriotypus, Archelirion, Martagon, Pseudolirium, Lilium, and Leucolirion (**Figure 3**). The morphological clustering results once again clearly demonstrated the uniqueness and diversity of all species in the Nomocharis clade.

### 3.3 Characteristics of Nomocharis clade chloroplast genome and ITS

In this study, we newly generated 21 complete chloroplast genomes in the genus *Lilium*, including 12 species from the Nomocharis clade [*L. gongshanense*, *L. saluenense*, *L. liangiae*, *Lilium medogense* S.Y.Liang, *Lilium souliei* (Franch.) Sealy, *Lilium paradoxum* Stearn, *L. meleagrinum*, *Lilium apertum* Franch., *Lilium sealyi* Y.D.Gao, *L. pardanthinum*, *Lilium yapingense* Y.D. Gao et X.J. He, and *L. nepalense* D.Don], two species from the Lophophorum clade [*Lilium nanum* Klotzsch in Klotzsch & Garcke and *Lilium lophophorum* (Bureau & Franchet) Franchet], and *Lilium wardii* Stapf ex F.C.Stern of the Sinomartagon clade. These sequences have been submitted to the GenBank database, and their accession numbers are listed in **Table 1**. These plastomes in the Nomocharis clade ranged from 151,735 bp (*L. pardanthinum*) to 152,915 bp (*L. nepalense*), exhibiting a typical circular quadripartite structure, composed of a large single-copy (LSC) region of 81,469–82,506 bp, a small single-copy (SSC) region of 17,031–17,616 bp, and two IR regions of 26,314–26,888 bp. The GC content of all these 21 chloroplast genomes was 37.0% (**Table 1**).

**Figure 4:**
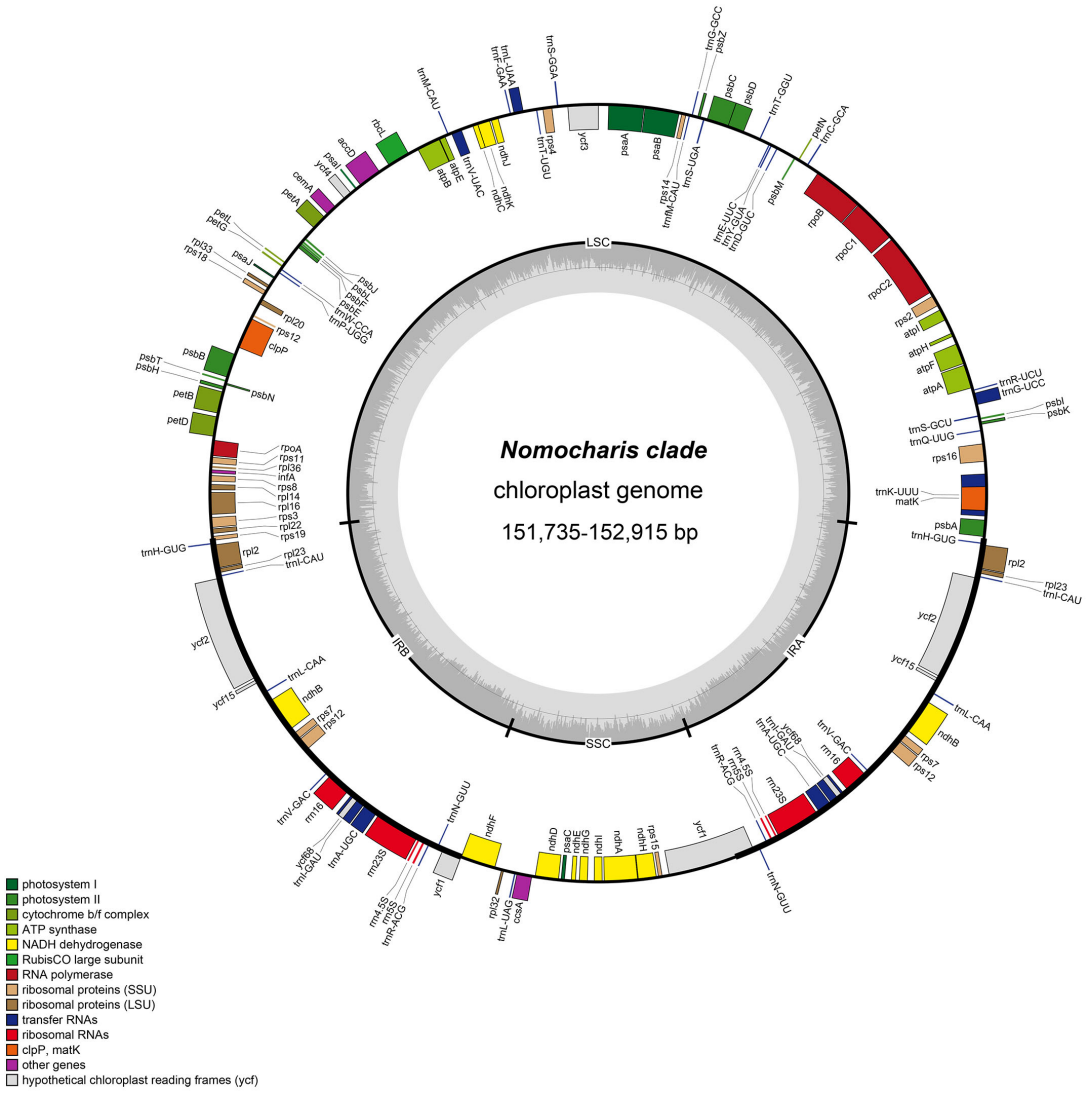
The chloroplast genome map of Nomocharis clade species. Genes of different functions were color-coded, with the inner circle showing G/C in dark gray and A/T in light gray. LSC, large single copy; SSC, small single copy; Ira and IRb, inverted repeats.

The gene content of the chloroplast genomes in the 21 samples is similar. Most of them are annotated to have 136 genes (115 unique), including eight rRNA genes (four unique), 38 tRNA genes (30 unique), and 84 protein-coding genes (78 unique) (**Tables 1**, **2**, **Figure 4**). Among these genes, six protein-coding genes (*rps12*, *ycf2*, *ndhB*, *rpl2*, *rps7*, and *rpl23*), four rRNA genes (*rrn23S*, *rrn16*, *rrn5S*, and *rrn4.5S*), and eight tRNA genes (*trnI-GAU*, *trnA-UGC*, *trnL-CAA*, *trnH-GUG*, *trnI-CAU*, *trnR-ACG*, *trnV-GAC*, and *trnN-GUU*) were duplicated due to being located in the IR region. Nine protein-coding genes (*atpF*, *ndhA*, *ndhB*, *petB*, *petD*, *rpl16*, *rpl2*, *rpoC1*, and *rps16*) contain one intron, and three protein-coding genes (*clpP*, *rps12*, and *ycf3*) contain two introns. Furthermore, in all newly generated *Lilium* chloroplast genomes, *rps12* has been identified as a noteworthy *trans*-splicing gene, with its 5′-exon located in the LSC region and the other end situated in the IRs (**Figure 4**). Four pseudogenes were identified, namely, *ψinfA*, *ψycf1*, *ψycf15*, and *ψycf68*, which were also found in other lilies and related genera (Du et al., 2017; Lu et al., 2017; Kim et al., 2019; Huang et al., 2020).

**Table 2:** Gene composition in *Lilium* chloroplast genome.

Group of genes	Name of genes
Photosystem I	*psaA*, *psaB*, *psaC*, *psaI*, *psaJ*
Photosystem II	*psbA*, *psbB*, *psbC*, *psbD*, *psbE*, *psbF*, *psbH*, *psbI*, *psbJ*, *psbK*, *psbL*, *psbM*, *psbN*, *psbT*, *psbZ*
Cytochrome	*ccsA*, *petA*, petB*, petD*, *petG*, *petL*, *petN*
ATP synthase	*atpA*, *atpB*, *atpE*, atpF*, *atpH*, *atpI*
NADH dehydrogenase	ndhA*, ndhB(×2)*, *ndhC*, *ndhD*, *ndhE*, *ndhF*, *ndhG*, *ndhH*, *ndhI*, *ndhJ*, *ndhK*
Rubisco	*rbcL*
RNA polymerase genes	*rpoA*, *rpoB*, rpoC1*, *rpoC2*
Proteins of small ribosomal subunit	*rps2*, *rps3*, *rps4*, *rps7(×2)*, *rps8*, *rps11*, rps12(×2)**, *rps14*, *rps15*, rps16*, *rps18*, *rps19*
Proteins of large ribosomal subunit	rpl2(×2)*, *rpl14*, rpl16*, *rpl20*, *rpl22*, *rpl23(×2)*, *rpl32*, *rpl33*, *rpl36*
rRNA	*rrna16(×2)*, *rrna23(×2)*, *rrna4.5(×2)*, *rrna5(×2)*
tRNA	trnA-UGC(×2)*, *trnC-GCA*, *trnD-GUC*, *trnE-UUC*, *trnF-GAA*, *trnG-UCC*, *trnG-GCC*, *trnH-GUG(×2)*, *trnI-CAU(×2)*, trnI-GAU(×2)*, trnK-UUU*, trnL-UAA*, *trnL-UAG*, *trnL-CAA(×2)*, *trnfM-CAU*, *trnM-CAU*, *trnN-GUU(×2)*, *trnP-UGG*, *trnQ-UUG*, *trnR-ACG(×2)*, *trnR-UCU*, *trnS-GCU*, *trnS-GGA*, *trnS-UGA*, *trnT-GGU*, *trnT-UGU*, trnV-UAC*, *trnV-GAC(×2)*, *trnW-CCA*, *trnY-GUA*
Maturase	*matK*
Membrane protein	*cemA*
Acetyl-CoA carboxylase	*accD*
Protease	clpP**
Hypothetical proteins and conserved reading frames	*ycf1*, *ycf2(×2)*, ycf3**, *ycf4*
Pseudogenes	*ψinfA*, *ψycf1*, *ψycf15(×2)*, *ψycf68(×2)*

*, genes with one intron; **, genes with two introns; (×2), genes with two copies.

In addition, 21 ITS sequences, from a total of 15 species (**Supplementary Tables S5**, **S9**), were generated, whose length ranged from 626 to 632 bp, Complete length of ITS sequence alignment with gaps was 636 bp and included 509 conserved sites (80.03%), 119 variable sites (18.71%), 74 parsimony-informative sites (11.64%), and 45 singletons (7.08%). The average G+C content in the ITS region was 60.36% (**Supplementary Table S9**). The mean genetic distance within the Nomocharis clade was notably low, at 3.59%, and even lower within the Nomocharis subclade, at just 0.27%. Moreover, the overall genetic diversity in the entire *Lilium* genus was also quite limited, at only 5.84% (**Supplementary Table S10**). The pattern of genetic distance in complete chloroplast genomes is similar and even lower. The overall mean distance among the 23 representative species from within the entire genus is 0.43% (**Supplementary Table S11**).

**Figure 5:**
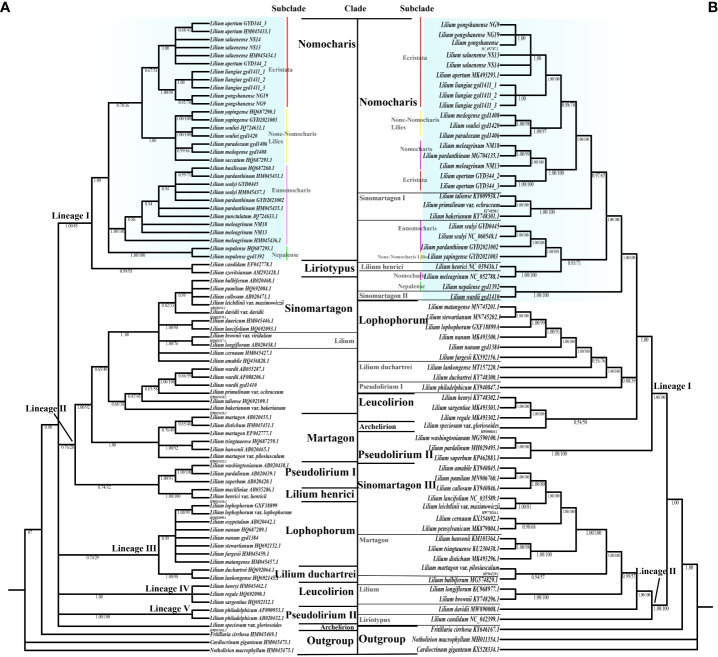
The phylogenetic relationships in the genus *Lilium* using Bayesian inference (BI) based on internal transcribed spacer (ITS). **(A)** Whole plastome sequence data. **(B)** Clade names based on Comber (1949), Liang (1980), and Gao et al.(2013). Bayesian posterior probabilities (PP) on left and bootstrap supports (BS) on right, and PP/BS = 1.00/100 not displayed. The Nomocharis clade is highlighted in blue, and its subclades are indicated.

The ITS and chloroplast genome lengths of the new species *L. liangiae* are 628 bp and 152,547–152,548 bp, with GC content of 60.35% and 37%, respectively (**Table 1**, **Supplementary Table S9**). Genetic distance and phylogenetic analysis indicate that this species is most closely related to *L. gongshanense* (**Figure 5**, **Supplementary Tables S10**, **S11**).

### 3.4 Phylogenetic relationships of *Lilium* genus

Previous molecular studies have already provided a reliable framework for the whole *Lilium* genus, but due to the endemicity of the Nomocharis clade to the H-D Mountains and the consequential lack of samples, there are still considerable insufficiencies in Nomocharis clade research. In our current study, the phylogenetic analysis is based on the complete chloroplast genome and nrITS respectively, including representative species in 12 clades (according to Comber, 1949; Liang, 1980; Gao et al., 2013) within the genus *Lilium* with three sequences from closely related genera as an outgroup, and revealed a similar topology with differences in some specific clades. All topologies consistently demonstrated the expected reciprocal monophyly between the genus *Lilium* and the outgroups (**Figure 5**; **Supplementary Figures S1**, **S2**).

In the ITS phylogenetic analysis (**Figure 5A**; **Supplementary Figure S1**), a total of 79 accessions and 59 taxa were used, and tree inference results from both ML and BI methods shared a consistent topology with only small differences in support values. Each clade forms well-defined monophyly in the Bayesian ITS tree, except the Sinomartagon and Pseudolirium clades, with the whole tree being mainly divided into five lineages (PP = 0.96). The Liriotypus clade is sister to the Nomocharis clade (PP = 1.00, BS = 87), which is comprised of four morphologically distinct subclades, including two traditional morphological subclades, Nomocharis and Ecristata, as well as two phylogenetically defined subclades, Non-Nomocharis Lilies and Nepalense. Subclade Ecristata was weakly supported to be a sister clade of subclade Non-Nomocharis Lilies (PP = 0.53, BS = 35). The new putative taxon *L. liangiae* belongs to the subclade Ecristata and is closest to *L. gongshanense*.

The phylogenetic relationships became significantly more intricate in the analysis of the chloroplast genomes of 65 accessions and 47 taxa (**Figure 5B**; **Supplementary Figure S2**). Topologies from the two methods were consistent. The Bayesian chloroplast genome tree was divided into two lineages, and most clades were not monophyletic. The Nomocharis clade contained species from the Sinomartagon clade, and their sisters were Lophophorum, *Lilium duchartrei* Franchet, and Leucolirion (PP = 1.00). The subdivision of the Nomocharis clade was not as clear as in the ITS dataset, as species from subclades Ecristata and Nomocharis clustered together. *L. liangiae* was closest to *L. gongshanense* and *L. saluenense* in the subclade Ecristata (PP = 1.00, BS = 100).

The phylogenetic trees based on ITS and chloroplast genomes exhibited conflicts. The ITS tree was clearer and aligned well with taxonomic clades, but it generally had low support values. In the chloroplast genome tree, certain species of the Sinomartagon clade were nested within the Nomocharis clade, while other clades show clear evolutionary relationships due to increased genetic variation information.

### 3.5 Ancestral state reconstruction

**Figure 6:**
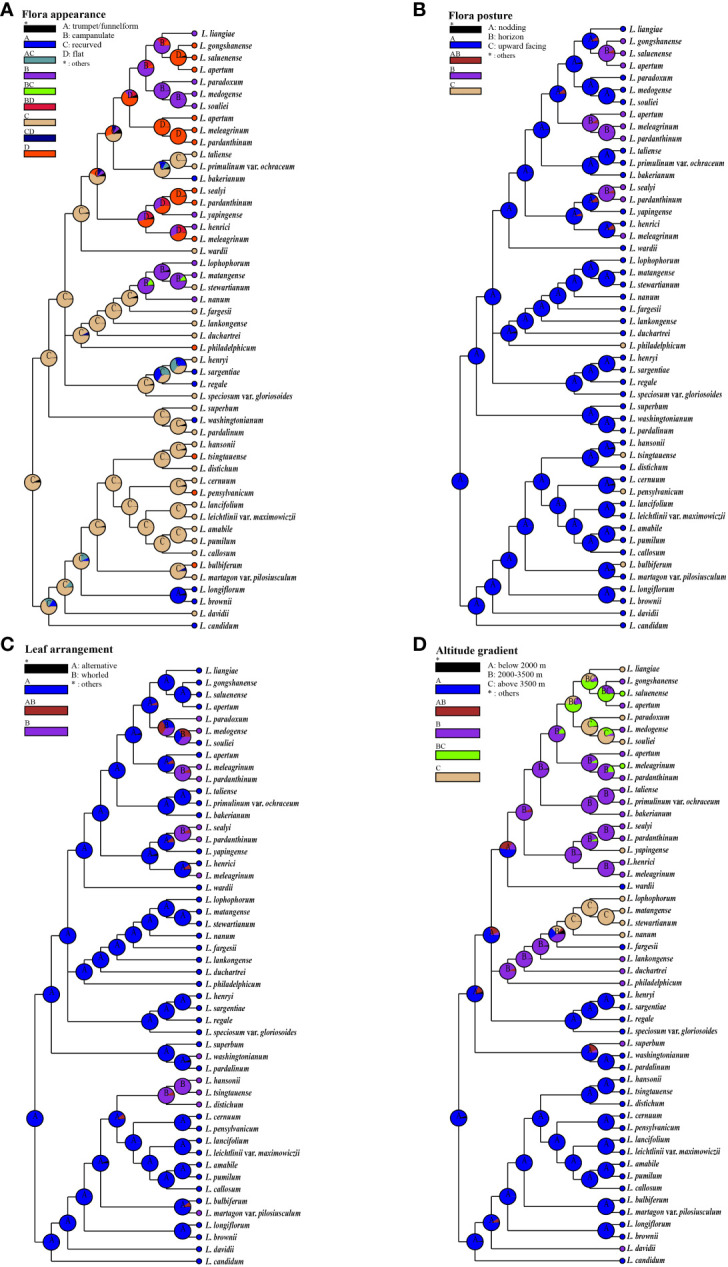
The ancestral state reconstructions of floral appearance, flora posture, leaf arrangement, and habitat altitude. Pie charts show probabilities of ancestral area reconstructions, and the most likely states are labeled in them. Colors of pie slices are defined in legend. Reconstructions of **(A)** flora appearance, **(B)** flora posture, **(C)** leaf arrangement, and **(D)** habitat altitude.

We conducted ancestral state reconstructions using a simplified chloroplast genome tree for four traits: flora appearance, flora posture, leaf arrangement, and habitat altitude (**Figure 6**; **Supplementary Table S7**). Our results indicate that recurved nodding flowers, alternate leaves, and low-altitude habitats (below 2,000 m) are most likely the ancestral conditions for lilies, this being in agreement with previous studies (Gao et al., 2015; Liu et al., 2019). There is a tendency for the nodding and campanulate features to be positively correlated. Additionally, we observed a disordered leaf arrangement, with whorled leaves arising at least four times within the genus *Lilium* (**Figure 6C**).

An association has been identified between the morphology of flowers and the altitude of their habitats. Features such as recurved tepals and trumpet-shaped nodding flowers, which are considered some of the most primitive traits, predominantly occur in regions situated at lower altitudes, specifically below 2,000 m. The evolution of upward-facing flat flowers presents a notably sporadic pattern across various altitudes, suggesting a recurrent and independent emergence of this trait. Moreover, the Nomocharis clade exhibits a distinct preference for mid- to high-elevation zones (2,000–3,500 m and above 3,500 m, respectively) through the prevalence of horizontal saucer-shaped flowers. Concurrently, species with bell-shaped nodding flowers demonstrate a proclivity for the higher elevation bands, being chiefly encountered above 3,500 m. This pattern denotes a potential adaptive significance in floral diversity corresponding to altitudinal variations.

### 3.6 Comparative analysis of plastome sequence

We conducted a comparison of the chloroplast genome boundaries among 23 species in the genus *Lilium*, with a primary focus on the species in the Nomocharis clade. Additionally, we selected one to two representatives from other clades, including clades Sinomartagon, Pseudolirium, Leucolirion, Liriotypus, Lophophorum, and Lilium duchartrei, for comparative analysis (**Supplementary Figure S3**; **Table S3**). The collinearity analysis and visual comparison of chloroplasts from 23 representative lily species revealed a high degree of sequence similarity, with no gene rearrangements observed (**Supplementary Figure S4**). The sequence variations were primarily concentrated at the conserved non-coding sequence (CNS) and *ycf1* and *ycf2* genes (**Supplementary Figure S5**). The findings also revealed slight variations in the expansion and contraction of the IRs. In all 21 chloroplast genomes, the boundaries of JSA (SSC-IRa) and JLA (IRa-LSC) exhibited a high level of consistency. The JSA boundary was identified inside the *ycf2* gene, extending from 1,102 to 1,656 bp into the IRa region, while the JLA boundary was positioned between trnH and psbA. The distance between the JLA boundary and psbA varied from 87 to 151 bp. The JLB (LSC-IRb) boundaries cut through the *rps19* gene, with 22 to 145 bp extending into the IRb region. Notably, there was a significant contraction of the JLB boundary in the IRb region (ranging from 22 to 31 bp) observed in seven *Nomocharis* species (*L. gongshanense*, *L. saluenense*, *L. medogense*, *L. paradoxum*, *L. sealyi*, *L. pardanthinum*, and *L. yapingense*). Regarding the JSB (IRb-SSC) boundary, which is located between or within the *ndhF* and *ycf1* genes, the *ndhF* gene was entirely present within the SSC region in most *Lilium* species except *L. gongshanense*, *L. saluenense*, *L. nanum*, *L. lophophorum*, *Lilium philadelphicum* L., *Lilium washingtonianum* Kellogg, and *Lilium regale* E.H.Wilson, where the gene *ndhF* extended into the IRb regions with lengths ranging from 1 bp (*L. lophophorum*) to 55 bp (*L. nanum*) due to the expansion of IRs, and the *ycf1* gene is mostly located in the IRb region with a length ranging from 1,102 to 1,289 bp.

### 3.7 Characteristics of repeat sequences

**Figure 7:**
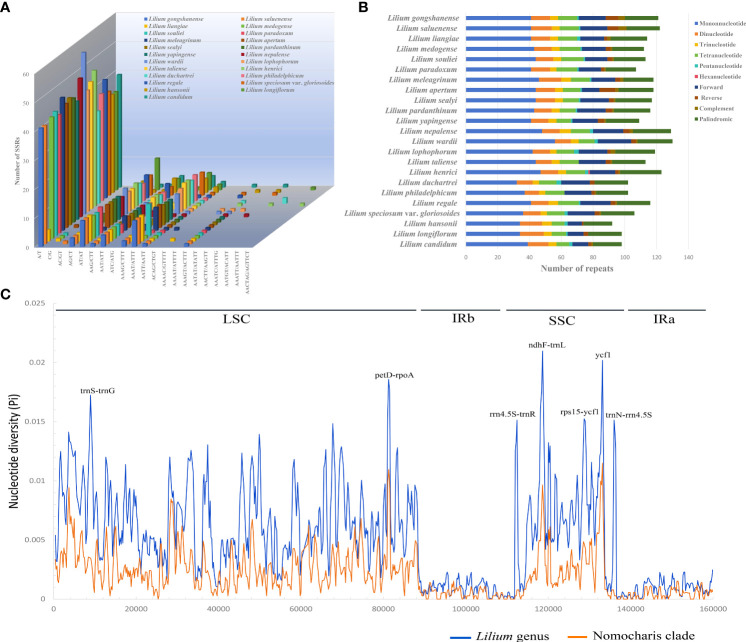
Patterns of repeat sequence **(A, B)** for the 23 chloroplast genomes of *Lilium* species and nucleotide diversity (Pi, C) for the genus *Lilium* (blue line) and Nomocharis clade (orange line). **(A)** Number of all kinds of simple sequence repeats (SSRs). **(B)** Number of SSRs, forward, reverse, complementary, and palindromic repeats. **(C)** Analysis of nucleotide diversity using 600-bp window length with a 200-bp step size. Seven high diversity regions (Pi ≥ 0.015) are indicated above the peaks, with the horizontal axis representing the window midpoints.

The number of SSRs in the 21 *Lilium* species ranged from 67 (*L. yapingense*) to 83 (*L. wardii*) (**Figures 7A, B**; **Supplementary Table S12**). The mononucleotide SSRs were the most abundant, with a count ranging from 41 to 56, while trinucleotide SSRs were the least abundant, only occurring four to seven times. Notably, among the SSR motifs, A/T, AT/AT, and AAAT/ATTT were the most frequently occurring repeats. Most notably, the A/T motif appeared in a range of 40 to 56 repetitions across all species, constituting over half of the total SSR count.

REPuter identified 36–50 long repeat sequences with a minimal repeat size set to 30 bp and a Hamming distance set to 3 (**Figure 7B**; **Supplementary Table S13**). These repeats included forward, reverse, complementary, and palindromic sequences. Palindromic repeat sequences were the most abundant, occurring 19 to 28 times, followed by forward repeats (11 to 21) and reverse repeats (2 to 10).

### 3.8 Molecular marker identification

We calculated nucleotide variability Pi (π) values for the whole *Lilium* genus (47 species) and separately for the Nomocharis clade (12 species) in the chloroplast genomes using sliding window analysis (**Figure 7C**; **Supplementary Tables S14**, **S15**). The whole *Lilium* genus analysis identified 3,092 singleton variable sites and 2,404 parsimony informative sites. A total of seven high nucleotide polymorphism regions (Pi ≥ 0.015), ranging from 695 to 1,157 bp, had been identified as potential genetic molecular markers, with the *petD*–*rpoA* intergenic spacer showing the highest mutations (Pi = 0.01857) in LSC and the *ndhF*–*trnL* intergenic spacer exhibiting the highest mutations (Pi = 0.02097) in SSC. The Nomocharis clade analysis exhibited a similar pattern, with significantly lower Pi values ranging from 0 to 0.01149 (*ycf1* gene).

## 4 Discussion

### 4.1 Morphological polymorphism and parallel evolution

Regarding the classification of clades within the *Lilium* genus, different authors hold varying perspectives, with the number of delineated clades ranging from seven to 16 (see table 1 in Watanabe et al., 2021), and we support dividing it into 11 clades based on morphology and existing molecular evidence, these being Sinomartagon, Lilium, Martagon, Pseudolirium, Lophophorum, Leucolirion, Liriotypus, Archelirion (Comber, 1949; Liang, 1980), Nomocharis, Lilium duchartrei, and Lilium henrici (Gao et al., 2013). Our phylogenetic framework based on the complete chloroplast genome and ITS was consistent with previous research. The Nomocharis clade is comprised of four subclades, namely, Nomocharis (equal to the former Eunomocharis), Ecristata, Non-Nomocharis Lilies, and Nepalense. The genetic distance among species within the entire Nomocharis clade is remarkably small, and polymorphism is clearly observed in the morphology of leaves, tepals, nectaries, and the entire plant. Even within the smallest subclades, certain essential traits continue to exhibit inconsistencies.

Subclades Nomocharis and Ecristata were originally groups when *Nomocharis* existed as an independent genus. They both exhibit the typical *Nomocharis* saucer-shaped features with distinct dark spots and blotches. The Nomocharis subclade, formerly named “Eunomocharis” by I.B. Balfour (1918), has nectaries at the base of inner tepals with flabellately arranged low ridges, filaments are swollen proximally or cylindrical, and most have whorled elliptic leaves. In the remaining three subclades, filaments are subulate, as is common in lilies generally. Nectaries of inner tepals in the subclade Ecristata species are comb-like and may be swollen on either side of the base or not (*L. gongshanense*). The Non-Nomocharis Lilies subclade now includes five species, *L. medogense*, *L. paradoxum*, *L. yapingense*, *L. souliei*, and *Lilium saccatum* S. Yun Liang, with campanulate nodding flowers, morphologically closer to the Lophophorum clade, and with no distinct nectaries. The Nepalense subclade only contains *L. nepalense*, which is characterized by a trumpet-shaped floral tube in which the tepals curl remarkably at the ends. Its pale yellow flowers, larger than those of the preceding three subclades, are morphologically closer to species within the other clades. The shape of its perianth cannot be simply described as “trumpet or funnelform” or “recurved”, so this species was excluded from morphological statistical analysis and ancestral state reconstructions (**Supplementary Tables S7**, **S8**).

The morphological polymorphism of lilies was considered the result of parallel evolution during rapid adaptation to various environments, especially for species located in the H-D Mountains and QTP (Liu et al., 2006; Sun et al., 2012; Zhang et al., 2014; Durán-Castillo et al., 2022; Barnbrook et al., 2023), where the Nomocharis clade was a typical example (Patterson and Givnish, 2002; Liu et al., 2006; Gao et al., 2012; Sun et al., 2012; Gao et al., 2013; Zhang et al., 2014; Gao et al., 2015; Givnish et al., 2020; Zhou et al., 2023). The observed relationship between floral morphology and altitude gradients (**Figure 6**) suggests a significant evolutionary response to environmental pressures. Initial morphologies such as recurved tepals and trumpet-form nodding flowers, which represent the most ancestral traits, predominate in lower elevation zones not exceeding 2,000 m. This distribution pattern might be linked to selective forces characteristic of low-altitude ecosystems.

In contrast, within the Nomocharis clade, there has been a discernible shift toward the prevalence of horizontal saucer-shaped flowers particularly adapted to mid- to high-altitude environments, spanning elevations from 2,000 up to 3,500 m, and even exceeding that threshold. Concurrently, bell-shaped nodding flowers seem to show a marked adaptation to high-altitude habitats, generally over 3,500 m. Such altitudinal differentiation in floral forms indicates a complex interaction between floral evolution and ecological adaptation, with varying morphologies reflecting distinct adaptive strategies to the unique conditions present at different elevation levels rather than being entirely correspondent to genetic relationships. Additionally, the multiple transformations in leaf arrangement also indicate that parallel evolution exists beyond floral structures. Although the specific mechanisms are unclear, this all suggests the ubiquity of parallel evolution within the genus *Lilium*, which warrants further investigation.

Our discovery of this new species again demonstrates the points mentioned above. *L. liangiae* possesses campanulate nodding flowers (a trait typical of the Lophophorum clade), purplish black nectaries with ridges of tissue arranged in a fan shape (a Nomocharis subclade typical trait), and tapered filaments at the lower end (a trait common to all *Lilium* species except the subclade Nomocharis), with phylogenetic results confirming its position within the Ecristata subclade of Nomocharis. The morphological complexity of *L. liangiae* connects the three subclades and the remaining *Lilium* clades. It also appears to downplay the significance of perianth morphology in traditional taxonomy, and we believe that environmental specificity is responsible for these, i.e., as a consequence of parallel evolution. In the field, *L. liangiae* struggles on a snowy mountain at over 3,500 m, coexisting with some herbaceous plants. Its bell-shaped, nodding flowers adeptly withstand precipitation, strong winds, and high levels of ultraviolet radiation. The observed polymorphism of filaments and nectaries raises questions about the pollinators involved and specific patterns within the entire Nomocharis clade.

### 4.2 Conflict in nuclear-plastid phylogenetic relationships

Regarding apparent phylogeny conflicts within the *Lilium* genus, clades appeared parallel in the ITS tree. With a ~600-bp data size, ITS effectively distinguishes species and genera but cannot elucidate their evolutionary relationships. The complete chloroplast genome addressed this issue, offering rich variation data revealing current *Lilium* species differentiation. However, these data reflected the evolutionary processes of the genes themselves, not the true evolutionary relationships of the species, so conflicts were inevitable. The ITS phylogeny can correspond well with morphology, while complete chloroplast genome phylogeny exhibited geographic preference. Hybridization and introgression now appear to be the primary causes of *Lilium* nuclear-plastid conflict (Gao et al., 2013, 2015, 2020; Zhou et al., 2023), the evidence consisted of the conspicuous geographical divisions observed in the plastid phylogeny corresponding to relatively distant species in the nuclear phylogeny, and it was not random (Gao et al., 2013, 2015).

Due to the relatively narrow distribution of species within the Nomocharis clade and the greater dispersal capability of pollen compared to seeds, hybridization was most likely to occur among species of *Nomocharis* and their close neighboring lilies. Our previous work (Gao et al., 2020) also examined three geographically close species, *L. gongshanense*, *L. meleagrinum*, and *L. saluenense*, within the Nomocharis clade, and found that they shared chloroplast genome types along the elevation gratitude. Although there was not sufficient evidence of hybrid speciation, continuous gene flow was found to be widespread across the nuclear genome, while the species identities are well maintained (Gao et al., 2020). Due to the immense genome of the *Lilium* species (44.88–167.58pg, Plant DNA C-values Database http://data.kew.org/cvalues/), the current sequence data quality and depth are insufficient to thoroughly address this issue. Further exploration and alternative methods are still needed.

### 4.3 Comparative analysis of the chloroplast genome

The 23 representative chloroplast genomes exhibited minimal variation in genome characteristics, such as length, the pattern of repeat sequences, gene content, and order, closely resembling those of other species within the Liliaceae family (Du et al., 2017; Lu et al., 2017; Kim et al., 2019; Huang et al., 2020; Wu et al., 2021; Duan et al., 2022; Sheikh-Assadi et al., 2022; Li et al., 2022b).

Repeat sequences and the expansion and contraction of IR boundaries play a crucial role in genome length variation (Ruhlman and Jansen, 2014; Zheng et al., 2017; Liao et al., 2021; Feng et al., 2022; Wang et al., 2022). In our study, the A/T motif and palindromic sequences are the most abundant in repeat sequences. The JLB (IRb-LSC) boundary intersects the *rps19* gene with a position difference of ~100 bp. The JSB boundary is typically found between the *ycf1* and *ndhF* genes, predominantly intersecting the *ycf1* gene. However, in the cases of *L. gongshanense*, *L. saluenense*, *L. washingtonianum*, and *Lilium candidum* L., especially in the first two species, the *ycf1* genes were significantly shorter. These variations result in ~100-bp differences in the length of the IRs. Potential molecular markers can significantly enhance research in species delimitation, phylogenetic analysis, and population genetics. We identified seven highly polymorphic regions (Pi ≥ 0.015) in this study, with most of them located in the SSC region. The regions *trnS*–*trnG*, *petD*–*rpoA*, *ndhF*–*trnL*, *rps15*–*ycf1*, and *ycf1* were reported in other lily species and related species of the genera *Fritillaria* and *Cardiocrinum* (Du et al., 2017; Lu et al., 2017; Kim et al., 2019; Huang et al., 2020; Sheikh-Assadi et al., 2022). These molecular markers were highly valuable for species delimitation, phylogenetic, and population genetic studies within the genus *Lilium*.

Overall, the *Lilium* chloroplast genome was highly conservative in both sequence and structures (**Figure 7C**; **Supplementary Figures S4**, **S5**). Variations were mainly concentrated in the CNS of the SSC and LSC regions, as well as the genes *ycf1* and ycf2, indicating rapid changes in these regions during evolution (Li et al., 2021). Our new taxon, *L. liangiae*, exhibits the closest sequence similarity and phylogenetic relationship to *L. gongshanense* and *L. saluenense*, but the number of reverse and complement, as well as A/T repeats, was significantly less than in the latter two (**Figures 5**, 7A, B). The Nomocharis clade, however, exhibits extremely low genetic variation in the chloroplast genome (the mean genetic distance is 0.22%, **Supplementary Table S11**), as well as in the chloroplast genome structure (**Supplementary Figures S4**, **S5**), and thus provides little distinctive information compared to the members outside of this clade. The low variation rate as well as the introgression nature of the genus *Lilium* poses greater challenges for resolving the phylogenetic relationships among species.

### 4.4 The classification perspective of *Lilium* genus in this study

Traditional morphological classifications may have involved the subjective weighting of certain important traits. This was why we initially considered that *L. liangiae* might belong to the Lophophorum clade, as a result of overestimating the importance of the appearance of the perigone. In this study, through hierarchical cluster analysis without weighting, it clustered within *Nomocharis*, and this is consistent with the phylogenetic results. This might indicate that, to some extent, a comprehensive study of morphological characteristics remains effective as an important weapon for understanding taxonomic relationships. However, we were still uncertain whether traits should be weighted, given that certain traits indeed exhibit differences in functions and variation rates, and morphological-based classification necessitated a more comprehensive anatomical dataset, encompassing additional traits that are still unclear and not well understood. The present study inspires us to believe that the shape of the nectaries is taxonomically significant for some species, and this feature has also been considered an important characteristic at the sectional level (Watanabe et al., 2021).

Objectively, the more molecular data we have, the closer we are to the nature of the species tree. However, due to the enormity of the *Lilium* genomes, the available effective nuclear markers are relatively limited currently. Therefore, whether through morphology or molecular methods, a deeper exploration of valuable information is necessary to identify universally applicable patterns, validated through multiple factors like geography and phenological data. However, in this genus, a classification based solely on morphological appearance can be less reliable and viewed with less confidence, especially when parallel evolution has been detected, the latter rendering genetic data more reliable and robust in resolving the real phylogeny in these cases. The genus *Lilium* exhibits a considerable degree of parallel evolution within morphology, which is a great challenge and difficulty for taxonomists.

## 5 Conclusion

In this study, we proposed *L. liangiae* and revealed the morphological diversity within the Nomocharis clade. These contradictions such as large differences in morphology but with low genetic divergence or vice versa may be attributed to the adaptation to different habitats. The phenomena of morphological characteristics evolving rapidly in response to the environment accompanied by extremely low genetic divergences reveal the special parallel evolution of lilies. It suggests that the time since the divergence of these species is relatively short and that their relationships may not be as strongly supported as those inferred from morphological features that have arisen through parallel evolution while adapting to similar or the same environments. Thus, to reveal the real status and position of any particular taxon, taxonomists need to employ and combine all available tools including molecular information to clarify the deep phylogenetic relationships while considering more phenotypic characteristics together to define the species boundaries and understand the evolutionary history of a particular group.

## Data availability statement

The datasets presented in this study can be found in online repositories. The names of the repository/repositories and accession number(s) can be found below: https://www.ncbi.nlm.nih.gov/genbank/, OR763325; https://www.ncbi.nlm.nih.gov/genbank/, OR763324; https://www.ncbi.nlm.nih.gov/genbank/, OR763328; https://www.ncbi.nlm.nih.gov/genbank/, OR763329; https://www.ncbi.nlm.nih.gov/genbank/, OR763315; https://www.ncbi.nlm.nih.gov/genbank/, 0R763316; https://www.ncbi.nlm.nih.gov/genbank/, OR763317; https://www.ncbi.nlm.nih.gov/genbank/, OR763313; https://www.ncbi.nlm.nih.gov/genbank/, 0R763318; https://www.ncbi.nlm.nih.gov/genbank/, OR763312; https://www.ncbi.nlm.nih.gov/genbank/, OR763327; https://www.ncbi.nlm.nih.gov/genbank/, OR763326; https://www.ncbi.nlm.nih.gov/genbank/, OR763319; https://www.ncbi.nlm.nih.gov/genbank/, OR763320; https://www.ncbi.nlm.nih.gov/genbank/, OR763321; https://www.ncbi.nlm.nih.gov/genbank/, OR763322; https://www.ncbi.nlm.nih.gov/genbank/, OR763323; https://www.ncbi.nlm.nih.gov/genbank/, OR763311; https://www.ncbi.nlm.nih.gov/genbank/, 0R763314; https://www.ncbi.nlm.nih.gov/genbank/, 0R763310; https://www.ncbi.nlm.nih.gov/genbank/, OR763309; https://www.ncbi.nlm.nih.gov/genbank/, OR353697; https://www.ncbi.nlm.nih.gov/genbank/, OR353696; https://www.ncbi.nlm.nih.gov/genbank/, OR353700; https://www.ncbi.nlm.nih.gov/genbank/, OR353701; https://www.ncbi.nlm.nih.gov/genbank/, OR353690; https://www.ncbi.nlm.nih.gov/genbank/, OR353691; https://www.ncbi.nlm.nih.gov/genbank/, OR353692; https://www.ncbi.nlm.nih.gov/genbank/, OR353688; https://www.ncbi.nlm.nih.gov/genbank/, OR353693; https://www.ncbi.nlm.nih.gov/genbank/, OR353687; https://www.ncbi.nlm.nih.gov/genbank/, OR353699; https://www.ncbi.nlm.nih.gov/genbank/, OR353698; https://www.ncbi.nlm.nih.gov/genbank/, OR350450; https://www.ncbi.nlm.nih.gov/genbank/, OR353684; https://www.ncbi.nlm.nih.gov/genbank/, OR350449; https://www.ncbi.nlm.nih.gov/genbank/, OR353694; https://www.ncbi.nlm.nih.gov/genbank/, OR353695; https://www.ncbi.nlm.nih.gov/genbank/, OR353686; https://www.ncbi.nlm.nih.gov/genbank/, OR353689; https://www.ncbi.nlm.nih.gov/genbank/, OR353685; https://www.ncbi.nlm.nih.gov/genbank/, OR350448.

## Author contributions

YY: Writing – original draft, Writing – review & editing. YG: Writing – original draft, Writing – review & editing.
