# Supplementary material for: Lilium liangiae, a new species in the genus Lilium (Liliaceae) that reveals parallel evolution within morphology
Source: Front Plant Sci. 2024 Mar 27;15:1371237. doi: 10.3389/fpls.2024.1371237 (PMC11004424; doi:10.3389/fpls.2024.1371237)

Supplementary Material

# 1 Supplementary Figures and Tables

**1.1 Supplementary Tables**

[see the excel file]

Table S1 GenBank accession for published ITS used in this study.

Table S2 GenBank accession for published complete chloroplast genome used in this study.

Table S3 GenBank accession number and sample code corresponding to each species in chloroplast comparative genomic analysis.

Table S4 Comparison of circumscriptions of *Lilium* sections between Watanabe et al. (2021) and the present study.

Table S5 GenBank accession for 21 newly generated ITS of *Lilium.*

TableS6 characters used for parallel evolution patterns of Lilium in this study

Table S7 Trait states used in ancestral state reconstruction

Table S8 38 Morphological characteristics within 10 groups of the *Lilium* genus.

Table S9 Characteristics of the 21 newly generated ITS for *Lilium*.

Table S10 Estimates of ITS evolutionary divergence (P-distance) between different clades and individuals*.*

Table S11 Estimates of complete chloroplast genome evolutionary divergence within 23 representative sequences in *Lilium*.

Table S12 Summary statistics of small sequence repeat in each species.

Table S13 Summary statistics of long sequence repeat in each species.

Table S14 Nucleotide variability values (Pi≥0.015) for the whole *Lilium* genus.

Table S15 Raw output of nucleotide variability values (Pi≥0.005) for the *Nomocharis* clade.

**1.2 Supplementary Figures**

[see the .tif file]

**Supplementary Figure 1**.Phylogenetic tree of *Lilium* ITS obtained using the Maximum Likelihood (ML), Branch numbers represent bootstrap supports (BS) values, with BS=1.00 not displayed.


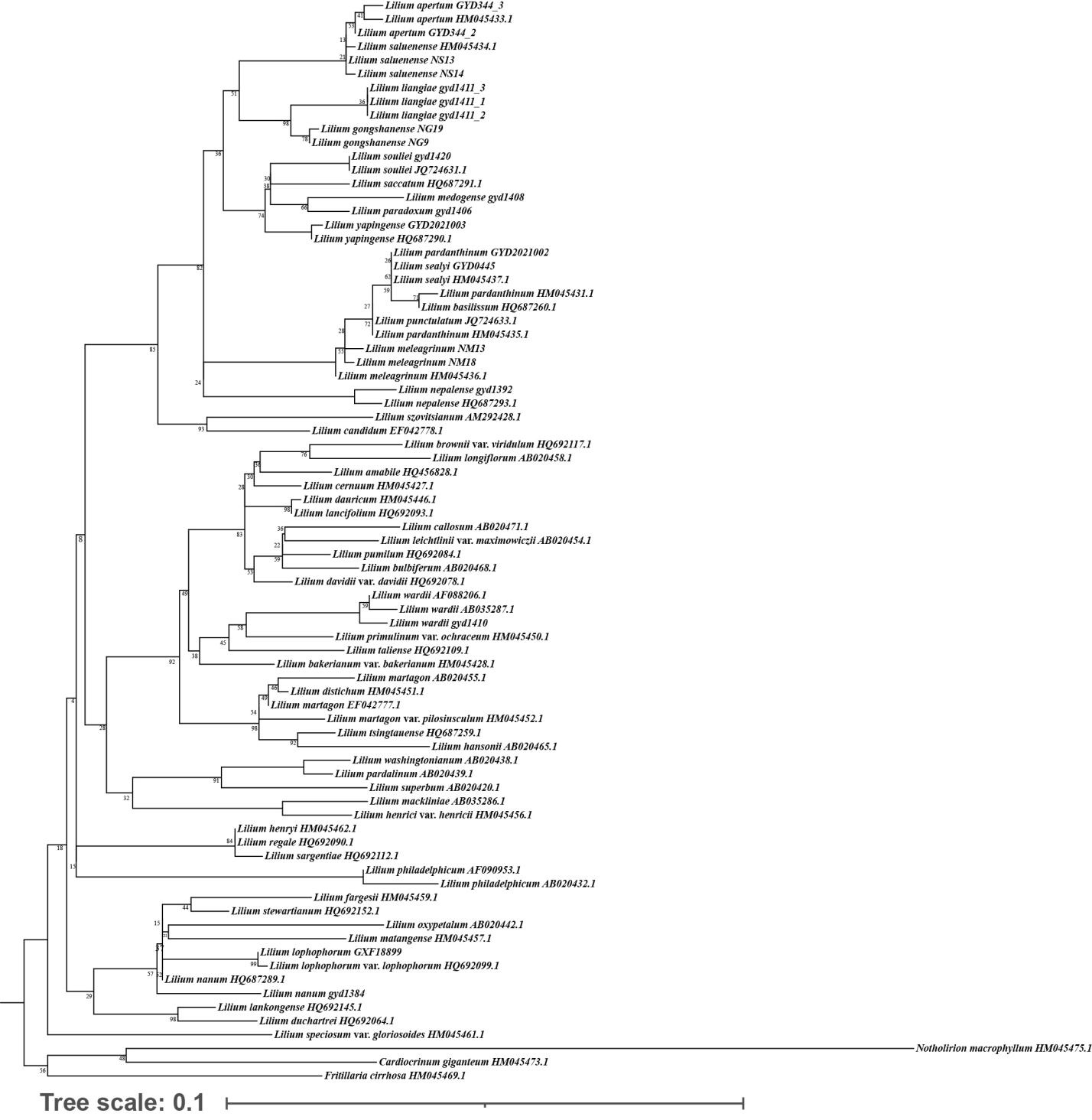


**Supplementary Figure 2.** Phylogenetic tree of *Lilium* chloroplast genome obtained using the Maximum Likelihood (ML), Branch numbers represent bootstrap supports (BS) values, with BS=1.00 not displayed.


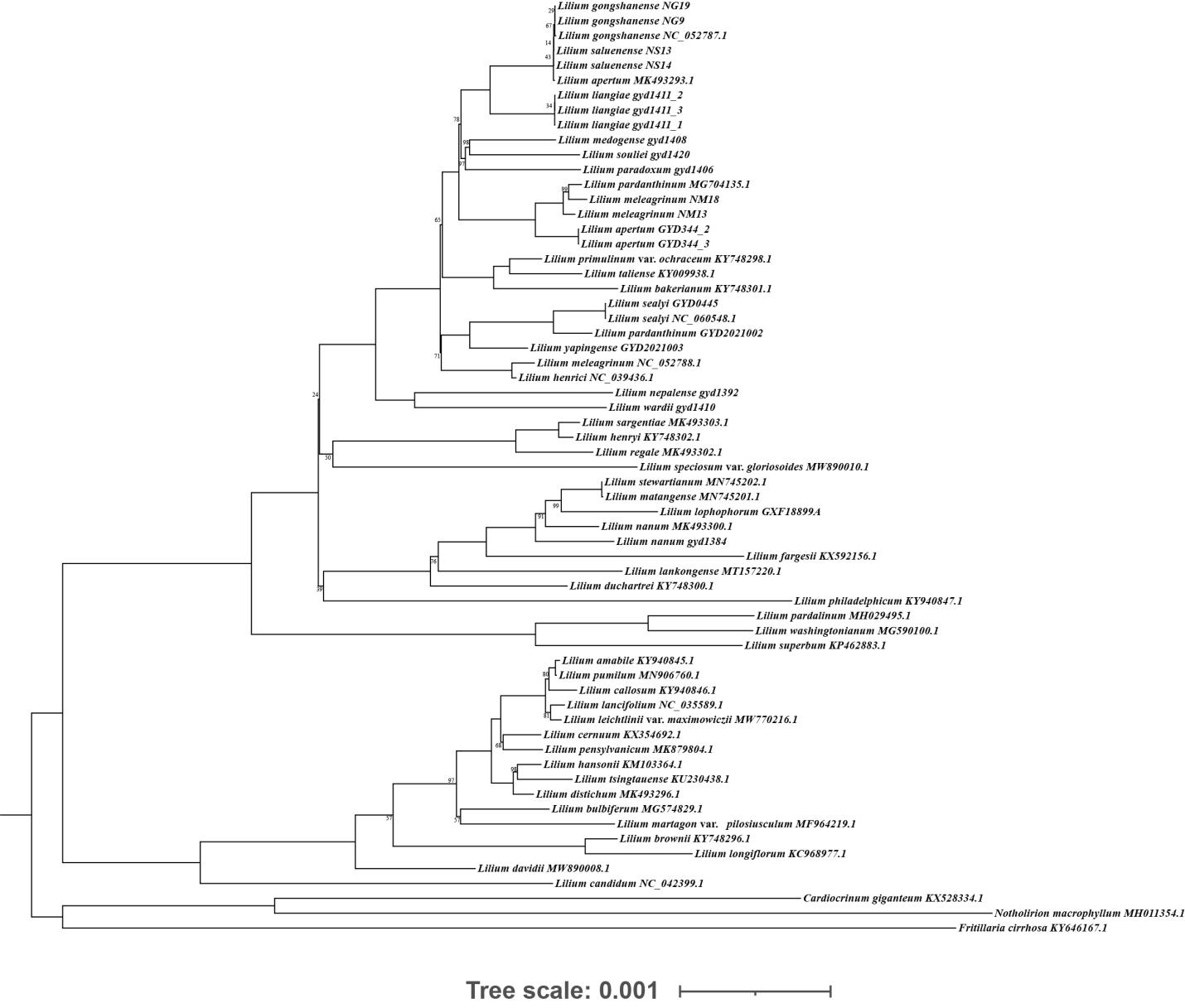


**Supplementary Figure 3.** Analyses of expansion and contraction of inverted repeats in the 23 *Lilium* chloroplast genomes.

**
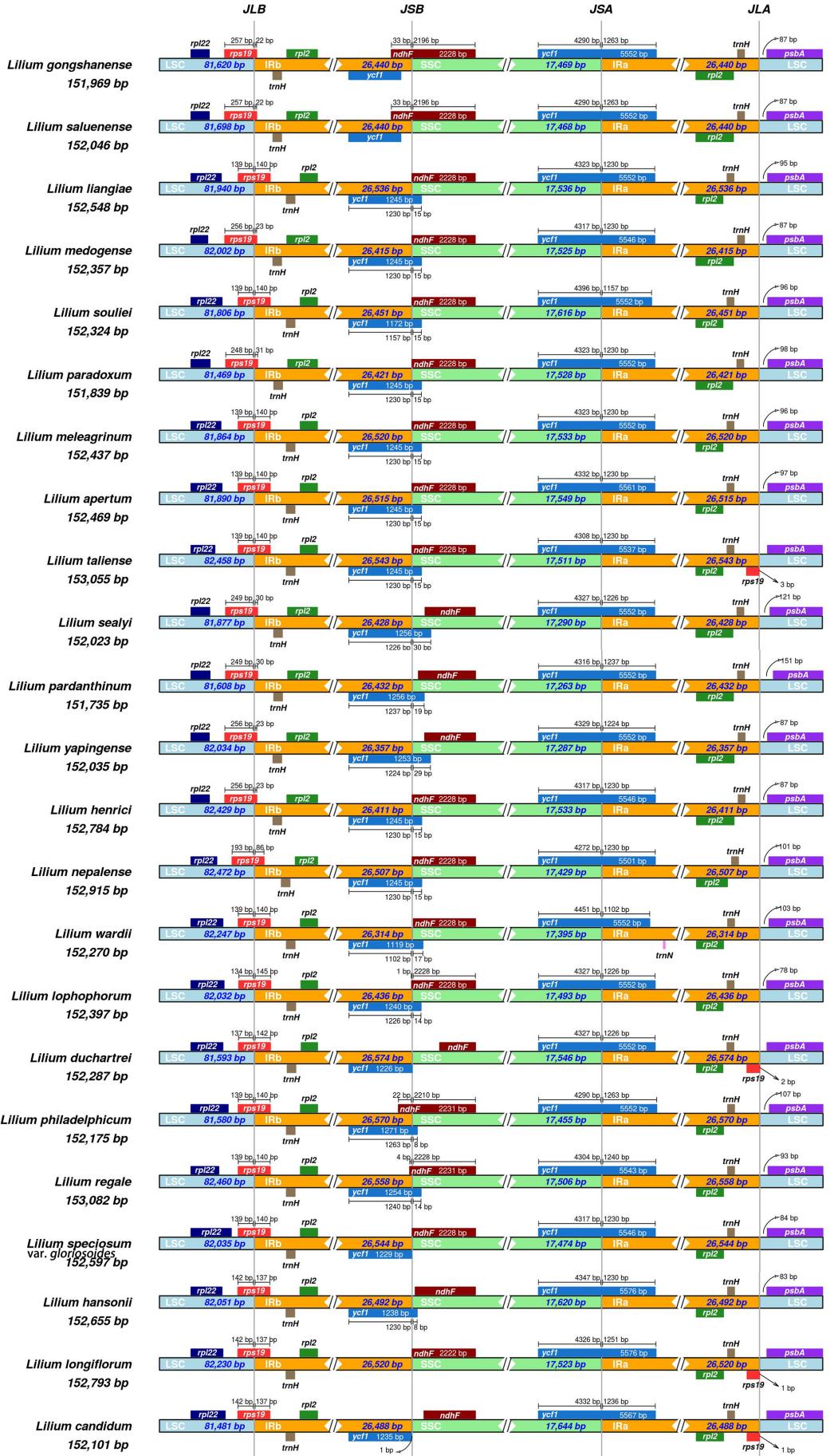
**

**Supplementary Figure 4**. Mauve alignment of 23 *Lilium* chloroplast genome revealing no interspecific rearrangements and the species name is at the bottom left of each sequence.


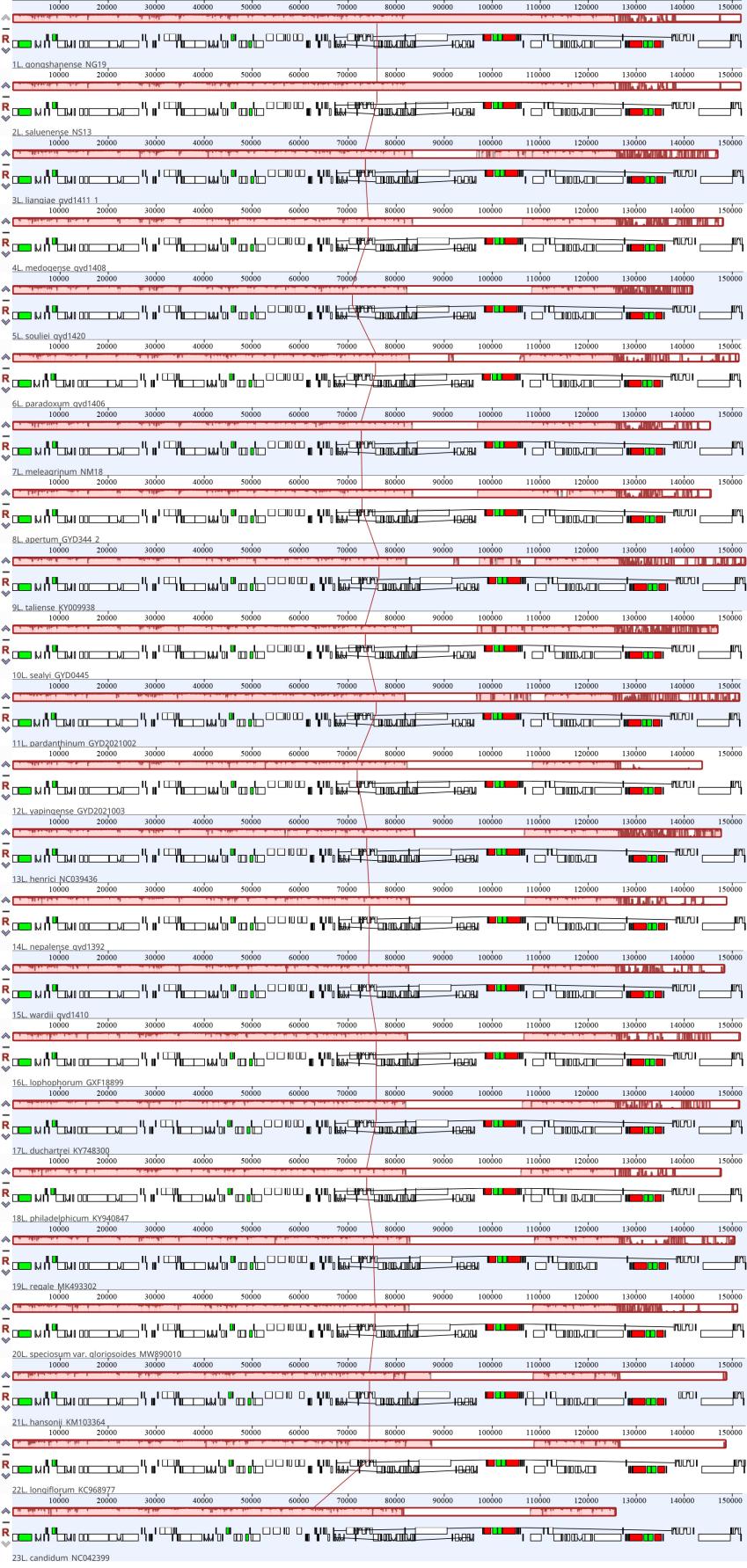


**Supplementary Figure 5**. Visualized alignment of the *Lilium* chloroplast genome sequences with annotated *L. gongshanense* as reference. The x-axis represents the position in the genome (kb), and the y-axis represents similarity. Legend displays sequence types, UTR stands for untranslated region, CNS stands for conserved noncoding sequence.


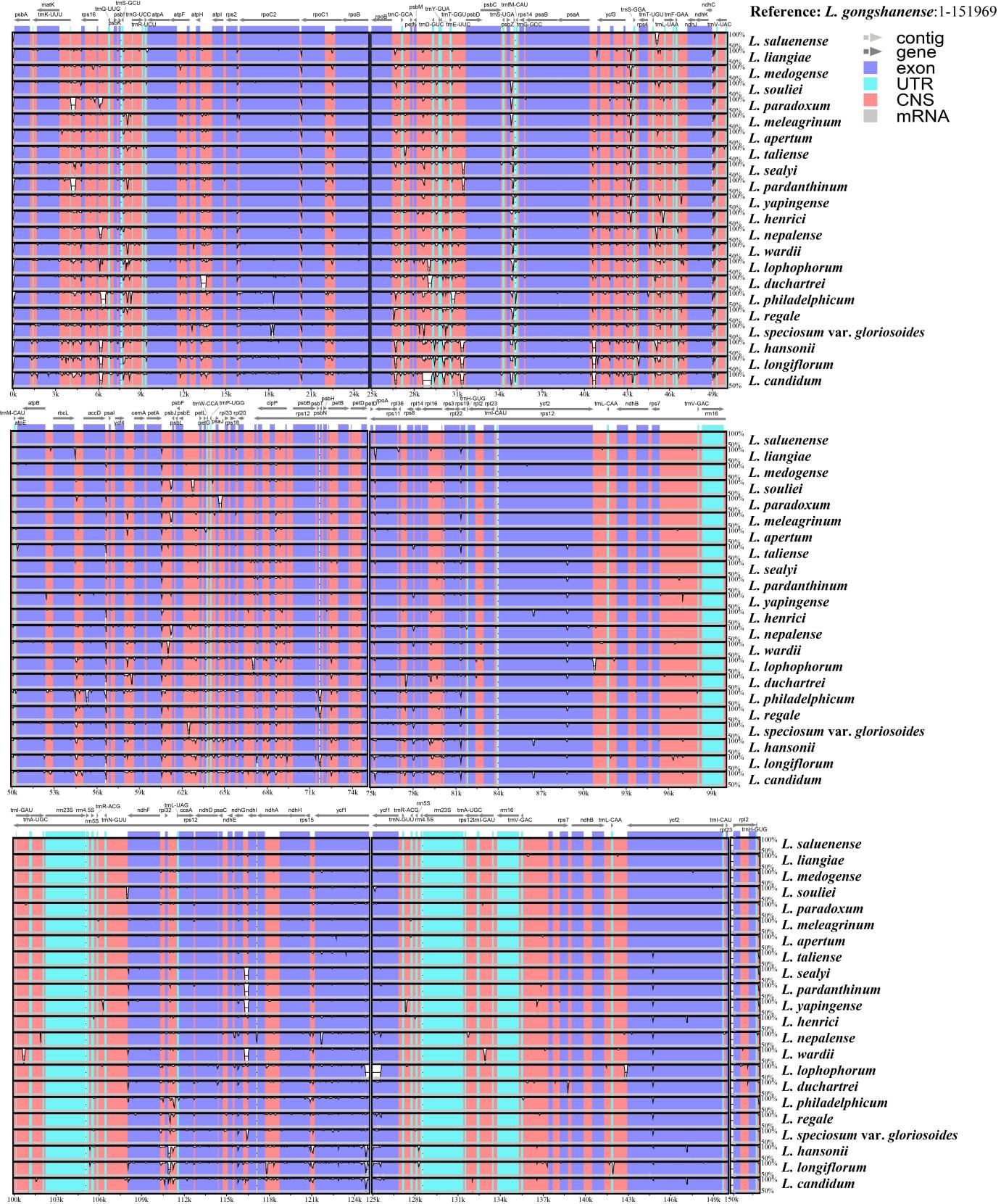

Supplement: Supplementary file 1 [file Table_1.docx]
